# Short-term consequences of F508del-CFTR thermal instability on CFTR-dependent transepithelial currents in human airway epithelial cells

**DOI:** 10.1038/s41598-019-50066-7

**Published:** 2019-09-24

**Authors:** Lionel Froux, Christelle Coraux, Edouard Sage, Frédéric Becq

**Affiliations:** 1 Laboratoire Signalisation et Transports Ioniques Membranaires (STIM), University of Poitiers, Poitiers, 86000 France; 2 Inserm UMR-S 1250, University of Reims Champagne-Ardenne, Reims, 51100 France; 3 Université Versailles-Saint-Quentin-en-Yvelines, Versailles, 78000 France; 4 Department of Thoracic Surgery and Lung transplantation, Hôpital Foch, Suresnes, 92150 France

**Keywords:** Electrophysiology, Chloride channels, Pharmacology, Cystic fibrosis, Cell culture

## Abstract

Loss-of-function mutations in the Cystic Fibrosis Transmembrane conductance Regulator (CFTR) channel in human airway epithelial cells are responsible for Cystic Fibrosis. A deleterious impact of physiological temperature on CFTR plasma membrane expression, residence and channel activity is characteristic of the most common and severe CF mutation, F508del. Using primary human F508del-airway epithelial cells and CF bronchial epithelial CFBE41o- cell lines expressing F508del- or WT-CFTR, we examined the effects of temperature (29 °C-39 °C) on the amplitude and stability of short-circuit CFTR-dependent currents over time and the efficiency of pharmacological strategies to stably restore F508del-CFTR function. We show that F508del-CFTR functional instability at 37 °C is not prevented by low temperature or VX-809 correction, genistein and VX-770 potentiators, nor by the combination VX-809/VX-770. Moreover, F508del-CFTR-dependent currents 30 minutes after CFTR activation at 37 °C did not significantly differ whether a potentiator was used or not. We demonstrate that F508del-CFTR function loss is aggravated at temperatures above 37 °C while limited by a small decrease of temperature and show that the more F508del-CFTR is stimulated, the faster the current loss happens. Our study highlights the existence of a temperature-dependent process inhibiting the function of F508del-CFTR, possibly explaining the low efficacy of pharmacological drugs in clinic.

## Introduction

The Cystic Fibrosis Transmembrane Regulator (CFTR) is a cAMP-dependent ligand-gated chloride channel belonging to the ATP-binding cassette (ABC) transporters family. CFTR is mainly expressed in secretory epithelia where it contributes to the regulation of ion and water transport^1^. In the lung, it plays a key role in hydration and pH regulation of airway secretions and loss-of-function mutations in the CFTR gene are responsible for Cystic Fibrosis (CF), the most common autosomal recessive disease among Caucasians^2^. CF is a life-shortening disorder affecting the function of multiple organs such as lungs, intestine, exocrine pancreas, biliary tree, sinuses and vas deferens, leading to a progressive multisystem failure in which loss of respiratory function and pancreatic insufficiency are the two major causes of morbidity^3^. Among the almost 2000 CF-causing CFTR variants, F508del is the most common^4^. It has multiple consequences on the CFTR protein: defective processing and intracellular transport leading to a reduced plasma membrane expression of the protein^5–8^, perturbation of channel gating leading to altered function^9^ and protein destabilization at the plasma membrane leading to an accelerated recycling and detection of the protein by the peripheral quality control^10–13^. A partial rescue of these multiple defects can be achieved *in vitro* by pharmacological approaches using the combination of a corrector (e.g. VX-809 and VX-661) and a potentiator (e.g. VX-770) to respectively enhance F508del-CFTR plasma membrane expression and function^14,15^. Such combinatorial approaches were recently approved by the Food and Drug Administration (FDA) for the treatment, *inter alia*, of patients homozygous for the F508del mutation. However, their clinical benefit on F508del-CFTR homozygotes remains under initial expectations^16^, suggesting that some defects caused by the F508del mutation might not be targeted by these therapies.

Several studies reported a deleterious impact of temperatures in the physiological range on F508del-CFTR plasma membrane expression^17,18^, single channel activity^19–21^ and plasma membrane residence of F508del-CFTR^22,23^. In accordance, plasma membrane expression and single channel activity of F508del-CFTR can partially be restored by placing cells at temperatures below 30 °C^12,17,20^. This suggests that temperature plays a central role in the appearance or at least the worsening of a majority of the defects caused by the F508del mutation. In this context, data from different groups demonstrated a run-down of F508del-CFTR function over a short time scale at physiological temperature^20,24–27^. Using diverse heterologous expression systems and electrophysiological methods such as patch clamp (single channel and whole cell configurations) and *Xenopus* oocyte whole cell recordings, they showed that most F508del-CFTR activity vanishes in less than 15 minutes. These observations raise the crucial question of the actual duration of F508del-CFTR activity during its residence at the plasma membrane. To date, all these studies were done in heterologous expression systems, often using methods in which intracellular solution is replaced by artificial solutions or plasma membrane isolated from the rest of the cell. It is then unclear if F508del-CFTR is behaving in the same way in a complex homologous system. The consequences on the ability of pharmacological strategies to restore a durable and stable function of F508del-CFTR are also unknown in this context.

Here, we combined the use of primary cultures of human airway F508del homozygous epithelial cells and human bronchial epithelial cell lines (CFBE41o^−^) expressing F508del- or WT-CFTR with Ussing chambers recordings, allowing a greater proximity from the physiological reality. In these conditions, we studied the effects of temperature on the amplitude and stability of CFTR-dependent currents over time and their consequences on the efficiency of pharmacological correctors and potentiators such as VX-809 (Lumacaftor) and VX-770 (Ivacaftor) to persistently restore F508del-CFTR function.

## Results

### CFTR-dependent current instability in primary cultures of human airway epithelial cells from F508del/F508del patients

**Figure 1:**
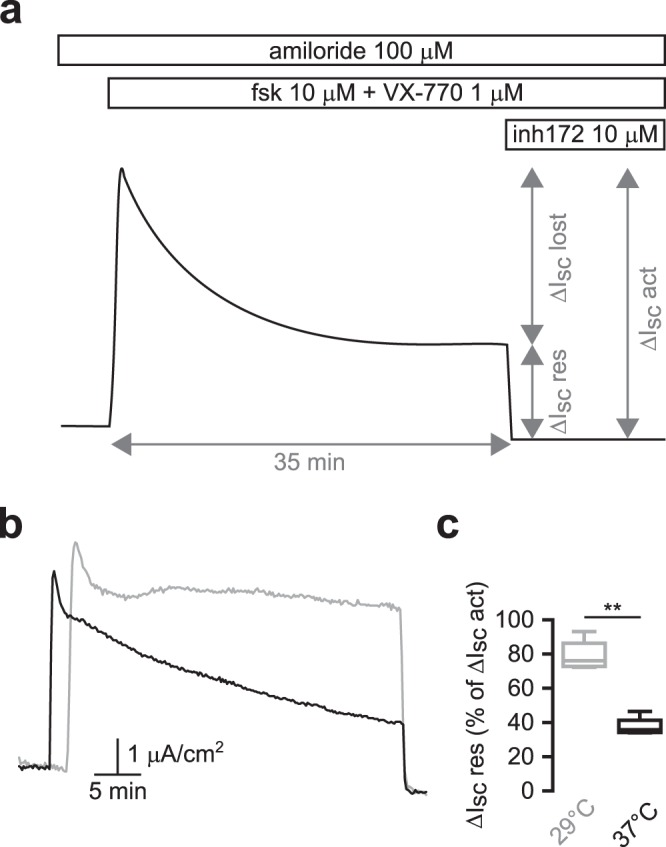
Functional thermal instability of CFTR-dependent transepithelial current in primary F508del/F508del HAE cells. (**a**) Schematic representation of a current obtained with the Ussing Chamber technique showing the parameters used to record and analyse CFTR-dependent I_sc_ stability over time. ΔI_sc_ res, residual ΔI_sc_ 35+/− 2 minutes after the peak or I_sc_ stabilization; ΔI_sc_ act, ΔI_sc_ after activation; fsk, forskolin; inh172, CFTR(inh)-172. (**b**) Representative Ussing Chamber recordings obtained in primary F508del/F508del HAE cells at 29 °C (grey line) or 37 °C (black line) with forskolin + VX-770 (1 μM) addition. (**c**) Box plot representations showing the distribution of ΔI_sc_ res values normalized to ΔI_sc_ act at 29 °C and 37 °C. **p < 0.01; two-tailed Mann-Whitney test; n = 5–6.

The principal aim of this study was to investigate the effect of physiological temperatures on F508del-CFTR-dependent transepithelial current in human airway epithelial (HAE) cells and the potential impact of temperature on F508del-CFTR function rescue by pharmacological potentiators. We first studied CFTR-dependent transepithelial current stability in primary HAE cells from F508del/F508del patients after low temperature correction (27 °C, 24 h). Using a rigorous control of apical and basal solutions temperatures, we recorded short circuit current variations (ΔI_sc_) at 29 °C and 37 °C during more than 30 minutes following CFTR activation with forskolin (10 μM) and VX-770 (1 μM). To quantify the impact of temperature on CFTR function, we focused on two important parameters reflecting CFTR function over time: the ΔI_sc_ after CFTR activation (hereafter noted ΔI_sc_ act) defined as the difference between I_sc_ at the peak or after stabilization and I_sc_ after CFTR(inh)-172 addition and the residual ΔI_sc_ after 35 minutes (hereafter noted ΔI_sc_ res) defined as the variation between I_sc_ 35 min after the peak or I_sc_ stabilization and I_sc_ after CFTR(inh)-172 addition (Fig. 1a).

The mean ΔI_sc_ act triggered by forskolin + VX-770 was 4.77 ± 0.76 μA.cm^−2^ (n = 5) at 29 °C and 7.24 ± 0.99 μA.cm^−2^ (n = 6) at 37 °C. We then observed a severe reduction of CFTR-dependent ΔI_sc_ during time at 37 °C (Fig. 1b) and mean ΔI_sc_ res represented only 37.65 ± 1.97% of ΔI_sc_ act (Fig. 1c). Conversely, CFTR-dependent transepithelial current was stable at 29 °C after an initial transitory phase (Fig. 1b) and mean ΔI_sc_ res represented 78.88 ± 3.77% of ΔI_sc_ act in this condition (Fig. 1c). These results demonstrate the instability of F508del-CFTR-dependent transepithelial current over time at physiological temperature in primary human CF airway epithelial cells.

### Recording CFTR-dependent current stability in CFBE cells

To investigate in detail the effect of physiological temperatures on CFTR-dependent currents in airway epithelial cells in the context of a pharmacological correction of CFTR membrane expression and function, we used the CF bronchial epithelial CFBE41o^−^ cell line expressing either F508del- or WT-CFTR (CFBE-F508del or CFBE-WT respectively). To restore detectable levels of CFTR protein at the plasma membrane, CFBE-F508del cells were incubated with VX-809 (10 μM) for 24 h prior to experiments. VX-809 was also added to the apical solution during Ussing chamber recordings to mimic therapeutic conditions.

**Figure 2:**
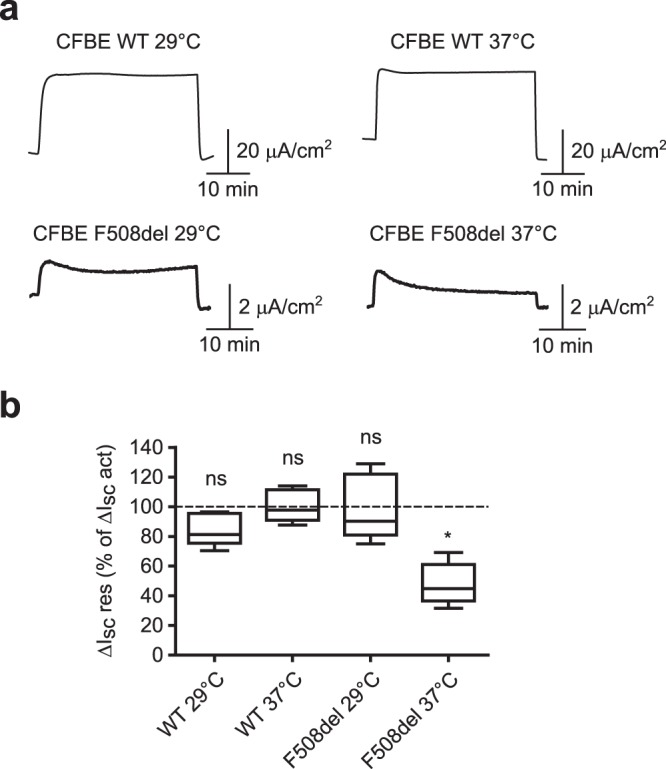
CFTR-dependent transepithelial current is not stable over time at 37 °C in CFBE-F508del cells. (**a)** Representative Ussing Chamber recordings obtained from CFBE-WT and CFBE-F508del cells at 29 °C or 37 °C after forskolin addition (10 μM). CFTR-dependent transepithelial current was inhibited with CFTR(inh)-172 addition (10 μM) after at least 35 minutes. VX-809 (10 μM) was added to the apical solution to mimic therapeutic conditions. (**b**) Box plot representations showing the distribution of ΔI_sc_ res values obtained in each condition presented in **a**. Data are expressed as the percentage of ΔI_sc_ act and tested for significant difference from 100%. *p < 0.05; ns, not significant; n = 5–6; Wilcoxon signed rank test. ΔI_sc_ res, residual ΔI_sc_ 35+/− 2 minutes after the peak or I_sc_ stabilization; ΔI_sc_ act, ΔI_sc_ after activation.

The current elicited by forskolin (10 μM) through CFBE-WT cells was systematically stable over the whole recording, whether solutions temperature was 29 °C or 37 °C (Fig. 2a, upper recordings). This was not the case with CFBE-F508del cells in which the forskolin-triggered current decreased rapidly at 37 °C to reach a plateau between 30 and 35 minutes, representing 47.81 ± 5.6% of the maximal current (significantly different from 100%; p = 0.031; n = 6; Wilcoxon signed rank test) (Fig. 2a, lower right trace). Conversely, CFTR-dependent current was stable over time in CFBE-F508del cells at 29 °C (Fig. 2a, lower left trace). Figure 2b shows the quantification and statistical analysis of ΔI_sc_ res for each experimental condition. These observations are thus comparable to what we measured using HAE cells from F508del/F508del patients.

In all cases the remaining current at the end of the recording was fully inhibited by CFTR(inh)-172 (10 µM), a selective blocker of CFTR^28^. These results show that the instability of F508del-CFTR function over time at physiological temperature described in other models^20,24–27^ is present and recordable in CFBE cells and confirm that VX-809 does not prevent the apparition of this phenomenon^27^.

Moreover, forskolin addition (10 μM) on non-corrected CFBE-F508del cells after pre-incubation with CFTR(inh)-172 (10 μM) did not affect I_sc_ baseline more than 0.14 ± 0.09 μA.cm^−2^ (n = 4) over 35 minutes, far below the I_sc_ variations recorded after CFTR stimulation (Supplementary Fig. S1). This confirms that Ussing Chamber recordings are stable enough to reliably evaluate I_sc_ stability over longer recordings than usual and that I_sc_ variations recorded in these conditions were fully CFTR-dependent.

### F508del-CFTR potentiation restores superior CFTR-dependent currents only during a short time period at 37 °C

To investigate the impact of F508del thermal instability on the ability of potentiators to rescue F508del-CFTR function in our model, we used VX-770, the sole FDA-approved CFTR potentiator and genistein, a widely used investigational CFTR potentiator. We recorded I_sc_ variations in CFBE-WT or CFBE-F508del cells at 29 °C and 37 °C following CFTR activation with forskolin (10 μM), forskolin + genistein (30 μM) or forskolin + VX-770 (1 μM).

**Figure 3:**
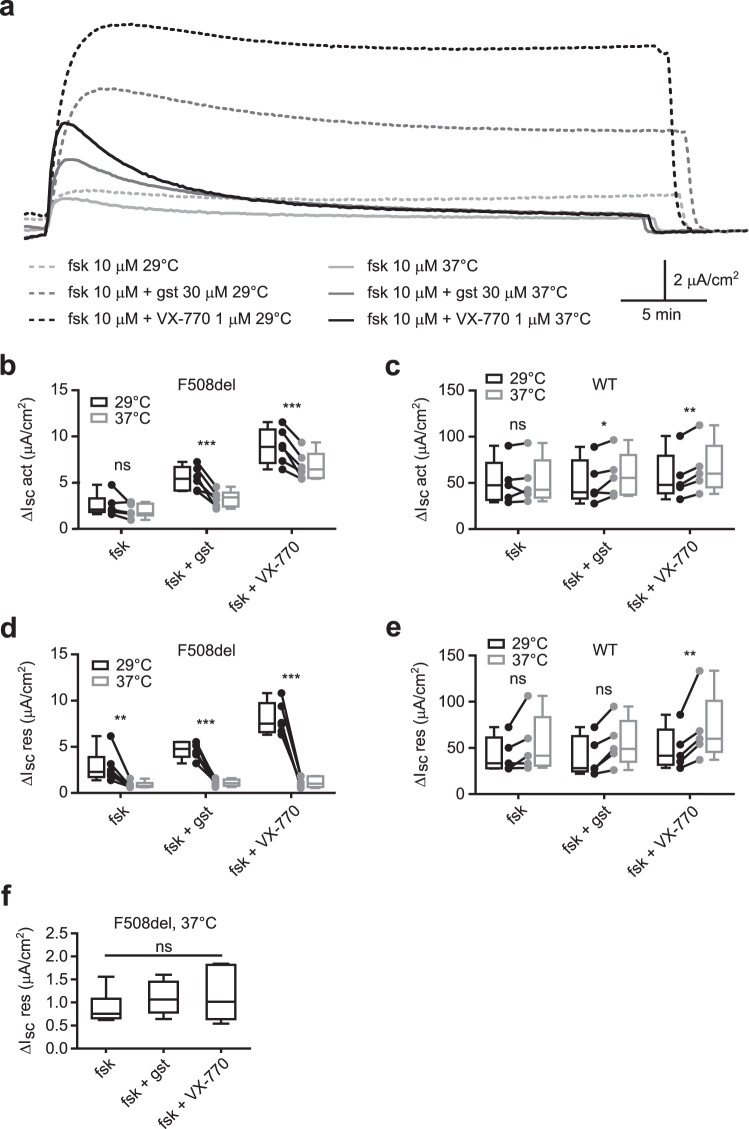
The beneficial action of classical CFTR potentiators vanishes over time at 37 °C in CFBE-F508del cells. (**a**) Ussing Chamber representative recordings obtained in CFBE-F508del cells at 29 °C (dotted lines) or 37 °C (plain lines) after forskolin (10 μM), forskolin + genistein (30 μM) or forskolin + VX-770 (1 μM) addition. VX-809 (10 μM) was added to the apical solution to mimic therapeutic conditions. (**b**,**c**) Graph displaying paired ΔI_sc_ act values obtained at 29 °C and 37 °C as well as their distribution, for forskolin, forskolin + genistein and forskolin + VX-770 conditions in CFBE-F508del (**b**) or CFBE-WT (**c**) cells. (**d**,**e**) Graph displaying paired ΔI_sc_ res values obtained at 29 °C and 37 °C as well as their distribution, for forskolin, forskolin + genistein and forskolin + VX-770 conditions in CFBE-F508del (**d**) or CFBE-WT (**e**) cells. (**a**–**e**) 29 °C and 37 °C recordings were systematically obtained on the same day on cells issued from the same passage for each condition. **p < 0.01; ***p < 0.001; ns, not significant; two-way repeated measures ANOVA and Bonferroni *post hoc* test (detailed results in supplementary table S5); n = 6. (**f**) Focus on the distribution of ΔI_sc_ res values obtained in CFBE-F508del cells with forskolin, forskolin + genistein and forskolin + VX-770. ns, not significant; Kruskal-Wallis test; p = 0.52; n = 6. ΔI_sc_ res, residual ΔI_sc_ 35+/− 2 minutes after the peak or I_sc_ stabilization; ΔI_sc_ act, ΔI_sc_ after activation; fsk, forskolin; gst, genistein.

In CFBE-F508del cells, when comparing ΔI_sc_ act values obtained at 29 °C with those obtained at 37 °C, we observed a significant decrease of this parameter with forskolin + genistein (5.49 ± 0.52 μA.cm^−2^ at 29 °C vs 3.29 ± 0.36 μA.cm^−2^ at 37 °C; n = 6; p = 0.0001; two-way repeated measures ANOVA and Bonferroni post-tests) as well as with forskolin + VX-770 (8.94 ± 0.77 μA.cm^−2^ at 29 °C vs 6.82 ± 0.61 μA.cm^−2^ at 37 °C; n = 6; p = 0.0002; two-way repeated measures ANOVA and Bonferroni post-tests), but no significant change with forskolin alone even if raw data showed a slight decrease in this condition (2.54 ± 0.47 μA.cm^−2^ at 29 °C vs 1.93 ± 0.30 μA.cm^−2^ at 37 °C; n = 6; p = 0.37; two-way repeated measures ANOVA and Bonferroni post-tests) (Fig. 3a,b). This represented a mean reduction of F508del-CFTR maximal function by 22 ± 7.26% with forskolin, 38.74 ± 6.41% with forskolin + genistein and 23.23 ± 3.52% with forskolin + VX-770 at physiological temperature. In comparison, ΔI_sc_ act was stable or slightly increased between 29 °C and 37 °C experiments in CFBE-WT cells, depending on the condition tested (n = 6 for each condition; p > 0.99 with forskolin, p = 0.046 with forskolin + genistein and p = 0.0094 with forskolin + VX-770; two-way repeated measures ANOVA and Bonferroni post-tests) (Fig. 3c). Thus, even if the effect of potentiators on F508del-CFTR maximal function is reduced at 37 °C compared to 29 °C, it still represents an improvement compared to forskolin stimulation alone. However, the improvement is not long-lasting.

Indeed, to take into account the thermal instability of F508del-CFTR function in our analysis, we focused on the residual current after 35 minutes, ΔI_sc_ res. As described above with forskolin stimulation alone, we found a marked instability of CFTR-dependent current with the two potentiators at 37 °C in CFBE-F508del cells (Fig. 3a, solid lines). At this temperature, the mean ΔI_sc_ res represented 47.81 ± 5.6% of the maximal current with forskolin, 34.13 ± 4.11% with forskolin + genistein and 16.53 ± 2.50% with forskolin + VX-770 (n = 6 for each condition) while I_sc_ values only slightly decreased over time at 29 °C (Fig. 3a, dashed lines). As a consequence, ΔI_sc_ res values obtained at 37 °C were considerably reduced compared to ΔI_sc_ res values obtained at 29 °C (n = 6 for each condition; p = 0.0049 with forskolin, p < 0.0001 with forskolin + genistein and forskolin + VX-770; two-way repeated measures ANOVA and Bonferroni post-tests) (Fig. 3d). CFTR-dependent current stability was not affected the same way in CFBE-WT cells and ΔI_sc_ res values were even significantly greater at 37 °C than at 29 °C in the forskolin + VX-770 condition (n = 6 for each condition; p = 0.19 with forskolin, p = 0.064 with forskolin + genistein and p = 0.0071 with forskolin + VX-770; two-way repeated measures ANOVA and Bonferroni post-tests) (Fig. 3e).

Moreover, if the two potentiators durably improved F508del-CFTR function compared to forskolin stimulation alone at 29 °C, this was not the case at 37 °C since there was no significant difference in ΔI_sc_ res between the three conditions (0.88 ± 0.14 μA.cm^−2^, 1.10 ± 0.15 μA.cm^−2^ and 1.15 ± 0.23 μA.cm^−2^ with forskolin, forskolin + genistein and forskolin + VX-770 respectively; n = 6; p = 0.52; Kruskal-Wallis test) (Fig. 3f). Therefore, the capacity of classical potentiators to enhance F508del-CFTR function is only transient at physiological temperature while it is stable over time at 29 °C.

### The rate of CFTR-dependent current loss over time is not affected by F508del-CFTR classical activators and potentiators

Since the residual current at 37 °C was the same whatever the way CFTR was stimulated while the maximal current differed depending on the activator cocktail used, we hypothesized that temperature-dependent current loss is a general phenomenon triggered upon CFTR activation and not affected by the way CFTR is activated.

**Figure 4:**
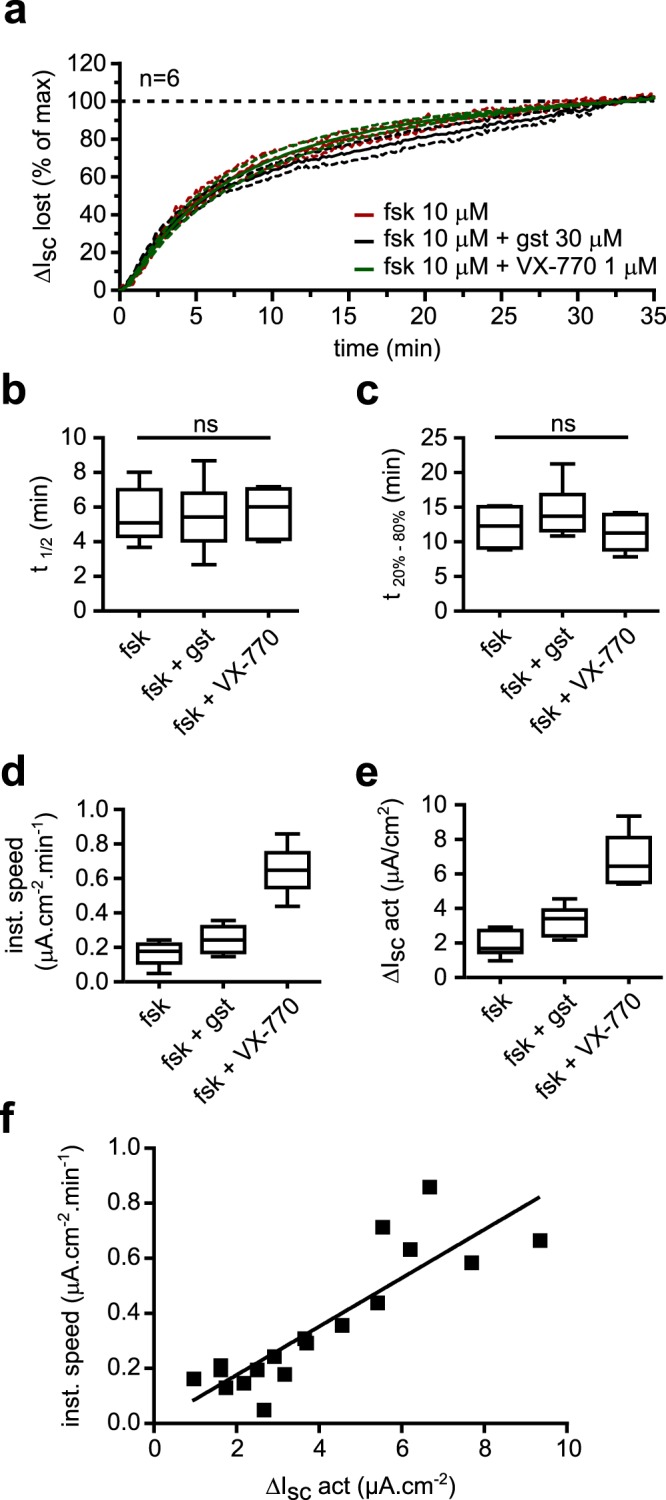
Current loss rate is not affected by CFTR potentiators but is correlated to CFTR-dependent maximal current. (**a**) Time course of the mean ΔI_sc_ lost expressed in % of maximal ΔI_sc_ lost against time for forskolin (10 μM), forskolin + genistein (30 μM) and forskolin + VX-770 (1 μM) conditions. (**b**) Corresponding t_1/2_ values distribution for each condition. Kruskal-Wallis test; p = 0.91; ns, not significant; n = 6. (**c**) Corresponding t_20%-80%_ values distribution for each condition. Kruskal-Wallis test; p = 0.29; ns, not significant; n = 6. (**d**). Distribution of instantaneous speed values 2 minutes after maximal ΔI_sc_ for forskolin alone, forskolin + genistein and forskolin + VX-770 conditions. n = 6. (**e**). Distribution of corresponding ΔI_sc_ act values in each condition. n = 6. **f**. Correlation of the instantaneous speed 2 minutes after maximal ΔI_sc_ and the ΔI_sc_ lost. Forskolin alone, forskolin + genistein and forskolin + VX-770 conditions were pooled and each square corresponds to a single trace. ΔI_sc_ act, ΔI_sc_ after activation; fsk, forskolin; gst, genistein.

If this is the case, the curves representing the normalized lost current plotted against time should be the same whatever the way F508del-CFTR was stimulated, while current loss speed should correlate with the maximal current amplitude. Using recordings presented in Fig. 3, we calculated the current loss evolution over time. Whether F508del was stimulated with forskolin (10 μM), forskolin + genistein (30 μM) or forskolin + VX-770 (1 μM), mean current loss curves were almost identical (Fig. 4a) and no significant differences were found between the three conditions for t_1/2_ or t_20%-80%_ (Fig. 4b,c). Reducing VX-770 or forskolin concentration did not significantly impact the current loss rate either (Supplementary Fig. S2). These results demonstrate that the way CFTR is activated does not impact the current instability.

Moreover, if we examine the instantaneous current loss rate at its maximal value (approx. 2 minutes after the maximal I_sc_ value) (Fig. 4d), we note that it evolves in the same way as the amplitude of ΔI_sc_ act (Fig. 4e), which was dependent on the way F508del-CFTR was activated. This suggested that the more F508del-CFTR was stimulated, the faster the current loss happened. Indeed, when plotting the instantaneous speed against ΔI_sc_ act, we observed a strong positive correlation between the two parameters (r = 0.87; p < 0.0001; n = 18) (Fig. 4f). These results strongly suggest that F508del-dependent current loss relies on an event intrinsically affecting the F508del-CFTR channel upon activation, independently of the way CFTR function is stimulated.

Several studies reported a negative impact of potentiators such as VX-770 on the correction of F508del-CFTR maturation defect by VX-809, suggesting an interference between potentiators and VX-809^29,30^. A negative interaction between VX-809 and potentiators could hence be responsible for the instability of F508del-CFTR-dependent currents observed at 37 °C during experiments presented above since VX-809 was added to the apical solution to mimic therapeutic conditions. In another set of experiments, we thus compared the stability of CFTR-dependent currents at 37 °C in CFBE-F508del cells with or without VX-809 in the apical solution (Supplementary Fig. S3). These experiments were realized with activation of CFTR by forskolin (10 μM), forskolin + genistein (30 μM) or forskolin + VX-770 (1 μM). No significant difference was observed between the two conditions concerning ΔI_sc_ act and t_1/2_ but we observed a significant augmentation of ΔI_sc_ res and t_20%-80%_, suggesting that VX-809 slightly limited F508del-CFTR-dependent current instability. These results show that VX-809 is not responsible for previously observed F508del-CFTR-dependent current instability at 37 °C.

### CFTR-dependent current instability dramatically worsens across a very small temperature range

**Figure 5:**
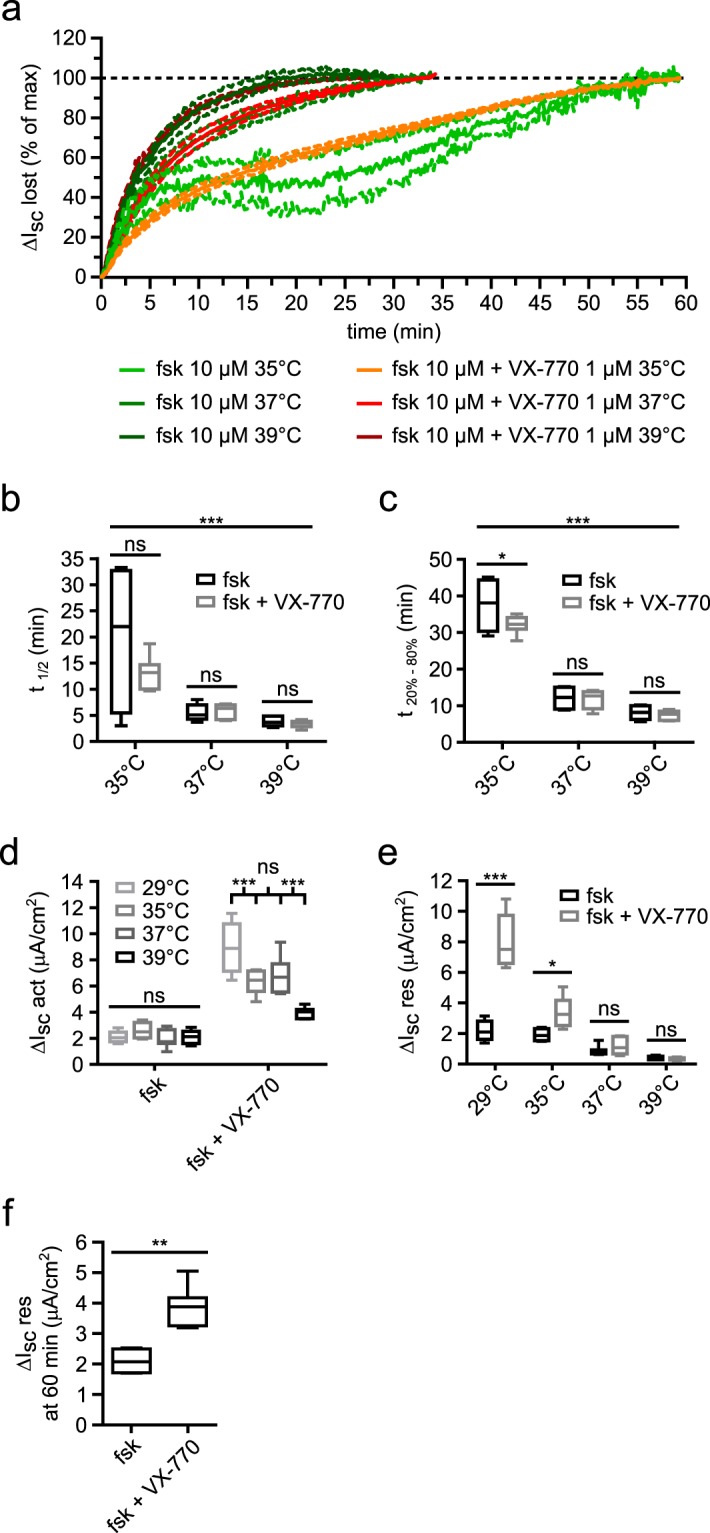
F508del-CFTR function loss at near-physiological temperatures. **(a**) Plot showing mean ΔI_sc_ lost expressed in % of maximal ΔI_sc_ lost against time for forskolin (10 μM) and forskolin + VX-770 (1 μM) conditions at 35 °C, 37 °C and 39 °C in CFBE-F508del cells. (**b**) Box plot representations showing the distribution of t_1/2_ values for each condition. ***p < 0.001; ns, not significant; two-way ANOVA and Bonferroni *post hoc* test; n = 4–7. (**c**) Box plot representations showing the distribution of t_20%-80%_ values for each condition. ***p < 0.001; *p < 0.05; ns, not significant; two-way ANOVA and Bonferroni *post hoc* test; n = 4–7. (**d**) Box plot representations showing the distribution of ΔI_sc_ act for forskolin and forskolin + VX-770 conditions at 29 °C, 35 °C, 37 °C and 39 °C in CFBE-F508del cells. ***p < 0.001; ns, not significant; two-way ANOVA and Bonferroni *post hoc* test; n = 4–7. (**e**) Box plot representations showing the distribution of ΔI_sc_ res for forskolin alone and forskolin + VX-770 conditions at 29 °C, 35 °C, 37 °C and 39 °C in CFBE-F508del cells. *p < 0.05; ***p < 0.001; ns, not significant; two-way ANOVA and Bonferroni *post hoc* test; n = 4–7. (**b**–**e**) Detailed results from two-way ANOVA analyses are summarized in Supplementary Table S5. (**f**) Box plot showing the distribution of residual ΔI_sc_ values 60 minutes after the peak for forskolin and forskolin + VX-770 conditions at 35 °C. **p < 0.01; two-tailed Mann-Whitney test; n = 4–7. ΔI_sc_ res, residual ΔI_sc_ 35+/− 2 minutes after the peak or I_sc_ stabilization; ΔI_sc_ act, ΔI_sc_ after activation; fsk, forskolin.

We also investigated the evolution of F508del current instability at temperatures surrounding 37 °C. Experiments presented in Fig. 3 were thus reproduced at 35 °C and 39 °C with forskolin alone and forskolin + VX-770. We noticed a considerable slowdown of F508del-CFTR-dependent current loss over time at 35 °C while it was accelerated at 39 °C (n = 4–7; p < 0.0001 for the temperature parameter; two-way ANOVA) (Fig. 5a–c). Since current loss was slowed down but not prevented by reducing the temperature to 35 °C, recordings were prolonged to 1 h to record a longer part of the current loss. At 39 °C, no differences in current loss kinetics between forskolin and forskolin + VX-770 conditions were observed. However, VX-770 addition induced a significant reduction of t_20%-80%_ but not t_1/2_ value at 35 °C compared to the forskolin alone condition (Fig. 5b,c). Given the high variability of the forskolin alone condition recordings, it only suggests that VX-770 might modestly accelerate F508del-CFTR-dependent current loss at colder temperatures.

Concerning current amplitudes, ΔI_sc_ act was not affected by temperature variation in the forskolin alone condition (Fig. 5d). In the forskolin + VX-770 condition, ΔI_sc_ act was stable between 35 °C and 37 °C but a further reduction of this parameter was observed at 39 °C. Indeed, mean ΔI_sc_ act value at 39 °C only represented 43.90% of mean ΔI_sc_ act value at 29 °C. This confirmed that forskolin-triggered F508del maximal I_sc_ was not sensible to temperature variations in the range studied here while potentiated F508del maximal I_sc_ was. Moreover, this sensitivity worsened dramatically when raising temperature above 37 °C.

ΔI_sc_ res was strongly reduced at 35 °C (57.50%) compared to 29 °C but was still higher than ΔI_sc_ res at 37 °C in the forskolin + VX-770 condition (Fig. 5e). In the forskolin alone condition, ΔI_sc_ res was stable between 29 °C and 35 °C but was strongly reduced between 35 °C and 37 °C (Fig. 5e). At 39 °C, ΔI_sc_ res was further reduced compared to 37 °C for both conditions. Of note, ΔI_sc_ res was almost two times higher with forskolin + VX-770 than with forskolin alone at 35 °C and this was still the case 1 h after forskolin + VX-770 addition (2.10 ± 0.22 μA.cm^−2^ with forskolin vs 3.85 ± 0.24 μA.cm^−2^ with forskolin + VX-770 at 37 °C; n = 4 and 7 respectively; p = 0.0061; two-tailed Mann-Whitney test) (Fig. 5f). Hence, VX-770 addition partially improved F508del-CFTR function over time at 35 °C, which was not the case at higher temperatures.

Together, these results show that F508del-CFTR function loss can be further aggravated at temperatures exceeding 37 °C while it can be limited by a small decrease of temperature, leaving a possibility for VX-770 to modestly improve F508del-CFTR function.

## Discussion

An increasing number of studies reported a thermal instability of F508del-CFTR function, leading to a rapid loss of F508del-CFTR current at physiological temperature^20,24–27^. However, the impact of F508del-CFTR thermal instability has not been assessed on CFTR-dependent transepithelial current in human airway epithelial cells. The principal aim of this study was thus to characterize the impact of F508del-CFTR functional thermal instability in primary HAE and CFBE cells during time and its potential consequences on the ability of potentiators to stably restore F508del-CFTR function in the context of a pharmacological correction with VX-809.

We demonstrate that F508del-CFTR thermal instability effects on CFTR-dependent transepithelial currents in human broncho-epithelial cells can be revealed using the Ussing chamber technique under particular conditions requiring a rigorous control of temperature. This defect was absent at 29 °C or in CFBE cells expressing WT-CFTR, demonstrating the existence of a deleterious impact of physiological temperatures on F508del-CFTR function over time in these models. Moreover, the resulting F508del-CFTR-dependent current loss was not prevented by VX-809 acute or chronic treatments and its kinetics was not affected by the way CFTR was stimulated. We also report a decrease in F508del-dependent current maximal amplitude at 37 °C compared to values obtained at 29 °C in CFBE cells, which was not the case with WT-CFTR.

When comparing the characteristics of F508del-CFTR current loss obtained here in CFBE cells with those reported previously in other models, we notice a high proximity despite models and technical approaches heterogeneity. Indeed, we report a 50% current loss in approximately 5–6 minutes depending on the condition tested, which is only slightly higher than the range of values previously reported (3 minutes in Wang *et al*.^20^; 4 minutes in Liu *et al*.^24^; 2–3 minutes in Boinot *et al*.^25^; 3–5 minutes in Meng *et al*.^27^).

Recently, Wang *et al*. concluded from single channel experiments that the potentiation of F508del-CFTR by VX-770 was temperature-independent at the single channel level^21^, since the maximal Po of each channel was the same below 30 °C and at 37 °C. However, our experiments show a decrease of F508del-dependent current maximal amplitude between 29 °C and 37 °C when a potentiator was used (genistein or VX-770). This apparent discrepancy could be explained by the fact that we recorded multiple CFTR channels, while the experiments realized by Wang *et al*. are single channel recordings. In our conditions, it is highly probable that some F508del-CFTR channels start to inactivate even during the global current increasing phase at 37 °C, which is not the case at 29 °C due to F508del-CFTR functional stability at this temperature. This can explain why a higher peak was observed at 29 °C compared to 37 °C in our conditions.

Despite the major reduction of F508del-dependent current amplitude over time, we observed a plateau with a residual CFTR(inh)-172-sensible current during each recording. This observation is in line with recent observations made by Meng *et al*., reporting the presence of a small subpopulation of F508del-CFTR channels characterized by a more stable function at physiological temperature in the presence of VX-809 and VX-770^27^. However, since the Ussing Chamber technique exclusively allows global current studies, this plateau could also be attributable, at least in part, to a residual activity at the single channel level.

Since most parameters of F508del-CFTR thermal instability described herein meet those reported previously by other groups using heterologous expression models and single channel or whole cell patch clamp recordings, our study also further confirms the efficiency of such approaches in predicting CFTR behaviour in models with higher physiological relevance, including primary HAE cells. Furthermore, given the similarity of our results with studies using excised membrane patches^24,27^, we can reasonably assume that the rapid thermal inactivation of F508del-CFTR channels is the major mechanism underlying the fast F508del-CFTR global current reduction described here at 37 °C.

As a consequence of F508del-CFTR functional thermal instability, we also demonstrate a considerable reduction of the beneficial effect of two CFTR potentiators across a short time range. Indeed, the residual current amplitude after more than 30 minutes does not differ whether a potentiator (VX-770 or genistein) was added or not to forskolin at 37 °C. Accordingly, the functional consequences of F508del-CFTR thermal instability imply the requirement of new stabilizing approaches to achieve a full and long-lasting restoration of F508del-CFTR function. These results could provide an explanation to the rather disappointing clinical results obtained with the co-administration of a “first-generation” corrector (VX-809 or VX-661) with VX-770 in F508del homozygotes despite an undeniable efficiency of VX-809 and VX-770 to respectively correct F508del-CFTR membrane expression and potentiate F508del-CFTR function (single channel conductance) *in vitro*^14–16,31^. However, a much better outcome on clinical signs in F508del homozygotes was reached during recent clinical trials led by Vertex Pharmaceuticals involving two triple combinations (VX-445 + VX-809 + VX-770 and VX-659 + VX-809 + VX-770) which integrate new correctors (i.e. VX-445 and VX-659), acting on F508del-CFTR through a different mechanism than VX-809^32,33^. Future studies are necessary to investigate if the improvement over FDA approved therapies can be explained by a potency of this new class of correctors to limit F508del-CFTR functional thermal instability.

Our results show that a small variation of temperature in the 35–39 °C range has a considerable impact on F508del-CFTR function. We could hypothesize that local temperature variations in lungs could significantly impact CF patients’ respiratory function. Supporting this idea, two recent studies explored the effect of temperature on CF lung disease in the US and Australia^34^ and in the general US population^35^, showing that patients residing in the warmest regions in the US have CF-specific lung function lower than if they resided in the coldest regions^34^. It was also reported a statistically significant association between warmer ambient temperature and reduced lung function in warmer regions, so that the lung function of individuals was 2% predicted lower on average than those living in colder regions^35^. In this regard, fever or local inflammation, two events frequently encountered by CF patients could also exacerbate CF symptoms.

The consequences of small temperature variations on F508del-CFTR function reported in the present study also recall the importance of an accurate temperature control during experiments assessing the function of F508del-CFTR and more generally channels with a function affected by temperature. Unfortunately, depending on the equipment and the technique used, maintaining extracellular temperature within 1 °C around the desired temperature can be very challenging. Efforts should thus be made to limit as much as possible temperature variations around the desired temperature during such experiments and technical limitations to do so should be kept in mind when interpreting results of F508del-CFTR functional studies. Of note, timing was also revealed to be an important parameter to consider in this context since F508del-CFTR function was considerably higher at the peak than 30 minutes after at physiological temperature.

More generally, taking into account F508del-CFTR thermal instability could help improving the detection of new molecules of interest correcting F508del-CFTR defects. For example, when questioning the capacity of molecules to individually restore F508del-CFTR plasma membrane functional expression (correctors) or channel activity (potentiators), setting bath temperature around 30 °C could be preferable to 37 °C since the thermal defect of F508del-CFTR could also impact transepithelial currents, introducing a potential bias. This would particularly be the case if temperature was not accurately controlled. On the other hand, when assessing the potential of molecules to correct the thermal stability defect, setting bath temperature at 37 °C seems critical.

Also, given the impact of F508del-CFTR thermal instability, the development of molecules able to stabilise F508del-CFTR function could be of interest to improve existing therapies. In this context, although they are not based on functional measurement of CFTR activity, the recent development of biophysical techniques such as the fluorescence self-quenching assay could be valuable to rapidly identify molecules able to correct F508del-CFTR thermal instability defect^36^. Ussing chambers or other function-based techniques amended for this purpose could then be used to confirm the potential of previously identified molecules to correct F508del-CFTR current instability at 37 °C.

In light of the results presented here and already reported by other groups^20,24–27^, taking into account the influence of temperature on F508del-CFTR function and other temperature-sensible mutants during future studies relating to the improvement of existing CF treatments or the development of novel ones could help improving their efficacy, likely leading to an improvement of CF patients’ quality of life.

## Methods

### Cell culture

Human airway epithelial (HAE) cells were obtained from explanted CF lungs at the time of patient’s transplantation. Human tissues were collected and used according to the French law, with the approval of the French Ministry of Higher Education and Research (Biological Collection n°DC-2012-1583) and with the informed consent of patients. Airways were dissected and epithelial cells obtained after overnight enzymatic dissociation using 0.5 mg/mL pronase E (Sigma Aldrich). HAE cells were seeded on type IV collagen-coated dishes and cultured in Pneumacul^™^-EX medium (StemCell Technologies) supplemented with tobramycin (80 μg.mL^−1^), ceftazidime (100 μg.mL^−1^), vancomycin (100 μg.mL^−1^), amphotericin B (0.25 μg.mL^−1^), penicillin (100 units/mL) and streptomycin (100 μg.mL^−1^). At confluence, cells were detached to be cultured at the air-liquid interface. Briefly, HAE cells from two distinct donors (P1) were seeded at a density of 0.1 × 10^6^ cells on type IV collagen-coated Transwell permeable inserts (#3801, Corning Corp.). They were cultured in liquid/liquid conditions in Pneumacult™-Ex medium (StemCell technologies) for 5–7 days until confluence was reached and then at air/liquid interface for 21 to 28 days using 1:1 DMEM/F12 (Gibco 31331) and BEGM (Lonza CC-3170) with the Lonza supplements for hEGF, epinephrine, BPE, hydrocortisone, insulin, triiodothyronine and transferrin, and supplemented with 50 IU.mL^−1^ penicillin (Sigma Aldrich), 50 μg.mL^−1^ of streptomycin (Sigma Aldrich), 0.1 nM retinoic acid (Sigma Aldrich) and 1.5 μg.mL^−1^ bovine serum albumin (Sigma Aldrich). Functionally detectable levels of F508del-CFTR were restored to the plasma membrane by incubating cells at 27 °C during 24 h prior experiments.

Human bronchial epithelial cell lines were provided by Dr. D. Gruenert (Univ. California San Francisco, USA). They were grown at 37 °C in 5% CO_2_ − 95% air and media were replaced every 2 days. CFBE41o- cells overexpressing F508del-CFTR (CFBE F508del-CFTR) or WT-CFTR (CFBE WT-CFTR) were grown on TPP^®^ culture flasks in Eagle’s Minimum Essential Medium (EMEM) containing non-essential amino acids (NEAA) (Gibco 10370) supplemented with 10% fetal bovine serum (FBS) (Sigma Aldrich), 2 mM L-glutamine (Gibco), 50 IU.mL^−1^ penicillin (Sigma Aldrich), 50 μg.mL^−1^ of streptomycin (Sigma Aldrich) and were selected using 5 μg.mL^−1^ puromycin (Gibco). CFBE CFTR-F508del and CFBE CFTR-WT cells were seeded at a density of 0.5 × 10^6^ cells on Snapwell permeable inserts (#3407, Corning Corp.) coated with 5 μg.cm^−2^ human fibronectin (Sigma Aldrich). After 2 days at liquid/liquid interface, cells were cultured at air/liquid interface until transepithelial resistance reached a minimum of 300 Ω.cm^−2^ (5–6 days). Transepithelial resistances were measured with a Millicell-ERS voltmeter-ohmmeter (Millipore). Functionally detectable levels of F508del-CFTR were restored to the plasma membrane by incubating cells with VX-809 (10 μM) for 24 h prior to experiments.

### Ussing chamber recordings

Inserts containing CFBE or primary HAE cells were mounted in an EM-CSYS-6 Ussing chamber system (Physiologic Instruments Inc., USA) composed of two hemi-chambers, each containing a different solution. Asymmetric solutions were used, creating a basal to apical Cl^−^ gradient to enhance Cl^−^ currents detection. Their composition was (in mM): 1.2 NaCl, 115 Na-Gluconate, 25 NaHCO_3_, 1.2 MgCl_2_, 4 CaCl_2_, 2.4 KH_2_PO_4_, 1.24 K_2_HPO_4_, 10 mannitol (pH 7.4) for apical solution and 115 NaCl, 25 NaHCO_3_, 1.2 MgCl_2_, 1.2 CaCl_2_, 2.4 KH_2_PO_4_, 1.24 K_2_HPO_4_, 10 glucose (pH 7.4) for basal solution. Apical and basal solutions were maintained at the desired temperature using a Julabo EC circulating temperature control water bath connected to the heat exchange block integrated into the Ussing chambers support and gassed with 95% O_2_ − 5% CO_2_. Transepithelial potential difference and short-circuit currents (clamp at 0 mV) were measured/injected through 3 M KCl filled Ag/AgCl electrodes connected to a VCC MC2 voltage/current clamp (Physiologic Instruments Inc., USA). The current injected by the system to short-circuit cells (I_sc_) was visualized and recorded at a frequency of 0.1 Hz on a personal computer using Acquire and Analyze hardware and software (Physiologic Instruments Inc., USA). Transepithelial potential difference values were corrected for the junction potential between apical and basal solutions and for empty insert resistance. In our conditions, an apical anion secretion was indicated by an increase in I_sc_. All experiments were done in the presence of amiloride (100 μM) in the apical solution to prevent ENaC currents. DIDS (4,4′-diisothiocyanatostilbene-2,2′-disulfonic acid; 200 μM) was added to basal and apical solutions to differentiate CFTR currents from most non-CFTR Cl^−^ currents^37–40^ except in experiments involving primary HAE cells where global transepithelial currents were studied. All other drugs were added to the apical solution. In all Ussing chambers experiments, I_sc_ value after CFTR(inh)-172 addition was used as the reference to calculate all ΔI_sc_ values presented in this article.

### Temperature control

Solution temperature was checked directly into each hemi-chamber of the Ussing chamber setup, as close as possible from the cells, with a digital precision thermometer (Checktemp^®^1, Hanna Instruments, USA) before and after each recording. For experiments at physiological or near physiological temperatures, results were discarded when temperature varied by more than 1 °C beyond or below the desired temperature. Experiments included in the 29 °C conditions were done at temperatures comprised between 27 °C and 31 °C. Supplementary Fig. S4 displays the distribution of the temperature values measured during all experiments presented in this article.

### Drugs and chemicals

Amiloride, CFTR(inh)-172, DIDS (4,4′-diisothiocyanatostilbene-2,2′-disulfonic acid), forskolin and genistein were purchased from Sigma Aldrich (France). VX-770 and VX-809 were obtained from Selleckchem (USA). Stock solutions (1000X) were prepared using dimethyl sulfoxide (DMSO) as solvent.

### Data analysis and statistics

Graphs and statistical analysis were done using Prism 5 (GraphPad Software, USA) and all the results are expressed as mean ± SEM except for box plot representations in which the central line represents the median, boxes upper and lower edges represent the 25th and 75th percentile respectively while the whiskers represent the minimal and maximal values of the sample. Statistical comparison were made with a significance threshold set at p < 0.05. In experiments comparing ΔI_sc_ values obtained at 29 °C with ΔI_sc_ values obtained at 37 °C (Fig. 3), 29 °C and 37 °C recordings were systematically obtained on the same day on cells issued from the same passage. Thus, a two-way ANOVA with repeated measures followed by a Bonferroni *post hoc* analysis has been used in this case. The same test was used during experiments comparing F508del-CFTR-dependent current stability with or without VX-809 in the apical solution (Supplementary Fig. 3). In other cases where multiple comparisons were required, two-way ANOVAs with a Bonferroni *post hoc* analysis or Kruskal-Wallis tests followed by Dunn’s multiple comparison tests were used depending on the number of parameters taken into account. A two-tailed Mann-Whitney test was used to compare data issued from two unpaired conditions. To improve readability, detailed results returned by two-way ANOVA tests presented in Figs 3 and 5 are summarized in Supplementary Table S5. The existence of a correlation between two parameters was tested by calculating the Pearson’s correlation coefficient and using a linear regression for graphical representation. In experiments focused on current loss, t_1/2_ and t_20%-80%_ represent the time required for CFTR-dependent current loss to reach 50% or to increase from 20% to 80% of its maximal value, respectively. Instantaneous speed 2 minutes after the peak was calculated using the following formula:

$$({\Delta {\rm{I}}}_{{\rm{sc}}{\rm{n}}+1}\,-\,{\Delta {\rm{I}}}_{{\rm{sc}}{\rm{n}}-1})/({{\rm{t}}}_{{\rm{n}}+1}-{{\rm{t}}}_{{\rm{n}}-1})$$

where ΔI_sc n+1_ and ΔI_sc n-1_ are the ΔI_sc_ values (μA.cm^−2^) surrounding the ΔI_sc_ value 2 minutes after the peak and t _n+1_ and t _n-1_ are the corresponding time values (min). Inst. speed 2 min is expressed in μA.cm^−2^.min^−1^.

## Supplementary information

Supplementary information

## Data Availability

The datasets generated during and/or analysed during the current study are available from the corresponding author on reasonable request.

## References

[1] Riordan JR (1993). The cystic fibrosis transmembrane conductance regulator. Annu. Rev. Physiol..

[2] Riordan JR (1989). Identification of the cystic fibrosis gene: cloning and characterization of complementary DNA. Science.

[3] Rowe SM, Miller S, Sorscher EJ (2005). Cystic Fibrosis. N. Engl. J. Med..

[4] Sosnay PR (2013). Defining the disease liability of variants in the cystic fibrosis transmembrane conductance regulator gene. Nat. Genet..

[5] Cheng SH (1990). Defective intracellular transport and processing of CFTR is the molecular basis of most cystic fibrosis. Cell.

[6] Denning GM, Ostedgaard LS, Welsh MJ (1992). Abnormal localization of cystic fibrosis transmembrane conductance regulator in primary cultures of cystic fibrosis airway epithelia. J. Cell Biol..

[7] Kartner N, Augustinas O, Jensen TJ, Naismith AL, Riordan JR (1992). Mislocalization of ΔF508 CFTR in cystic fibrosis sweat gland. Nat. Genet..

[8] Thomas PJ, Shenbagamurthi P, Sondek J, Hullihen JM, Pedersen PL (1992). The cystic fibrosis transmembrane conductance regulator. Effects of the most common cystic fibrosis-causing mutation on the secondary structure and stability of a synthetic peptide. J. Biol. Chem..

[9] Dalemans W (1991). Altered chloride ion channel kinetics associated with the ΔF508 cystic fibrosis mutation. Nature.

[10] Lukacs GL (1993). The delta F508 mutation decreases the stability of cystic fibrosis transmembrane conductance regulator in the plasma membrane. Determination of functional half-lives on transfected cells. J. Biol. Chem..

[11] Gentzsch M (2004). Endocytic trafficking routes of wild type and ΔF508 cystic fibrosis transmembrane conductance regulator. Mol. Biol. Cell.

[12] Sharma M (2004). Misfolding diverts CFTR from recycling to degradation: quality control at early endosomes. J. Cell Biol..

[13] Okiyoneda T (2010). Peripheral protein quality control removes unfolded CFTR from the plasma membrane. Science.

[14] Van Goor F (2009). Rescue of CF airway epithelial cell function *in vitro* by a CFTR potentiator, VX-770. Proc. Natl. Acad. Sci..

[15] Van Goor F (2011). Correction of the F508del-CFTR protein processing defect *in vitro* by the investigational drug VX-809. Proc. Natl. Acad. Sci..

[16] Wainwright CE (2015). Lumacaftor–Ivacaftor in Patients with Cystic Fibrosis Homozygous for Phe508del. CFTR. N. Engl. J. Med..

[17] Denning GM (1992). Processing of mutant cystic fibrosis transmembrane conductance regulator is temperature-sensitive. Nature.

[18] Rabeh WM (2012). Correction of Both NBD1 Energetics and Domain Interface Is Required to Restore ΔF508 CFTR Folding and Function. Cell.

[19] Hegedűs T (2006). F508del CFTR with two altered RXR motifs escapes from ER quality control but its channel activity is thermally sensitive. Biochim. Biophys. Acta BBA - Biomembr..

[20] Wang W, Okeyo GO, Tao B, Hong JS, Kirk KL (2011). Thermally Unstable Gating of the Most Common Cystic Fibrosis Mutant Channel (ΔF508): “Rescue” by Suppressor Mutations in Nucleotide Binding Domain 1 and by Constitutive Mutations in the Cytosolic Loops. J. Biol. Chem..

[21] Wang Y, Cai Z, Gosling M, Sheppard DN (2018). Potentiation of the cystic fibrosis transmembrane conductance regulator Cl^−^ channel by ivacaftor is temperature independent. Am. J. Physiol.-Lung Cell. Mol. Physiol..

[22] Sharma M, Benharouga M, Hu W, Lukacs GL (2001). Conformational and Temperature-sensitive Stability Defects of the ΔF508 Cystic Fibrosis Transmembrane Conductance Regulator in Post-endoplasmic Reticulum Compartments. J. Biol. Chem..

[23] Jurkuvenaite A (2010). Functional Stability of Rescued ΔF508 Cystic Fibrosis Transmembrane Conductance Regulator in Airway Epithelial Cells. Am. J. Respir. Cell Mol. Biol..

[24] Liu X, O’Donnell N, Landstrom A, Skach WR, Dawson DC (2012). Thermal Instability of ΔF508 Cystic Fibrosis Transmembrane Conductance Regulator (CFTR) Channel Function: Protection by Single Suppressor Mutations and Inhibiting Channel Activity. Biochemistry.

[25] Boinot C, Jollivet Souchet M, Ferru-Clement R, Becq F (2014). Searching for Combinations of Small-Molecule Correctors to Restore F508del-Cystic Fibrosis Transmembrane Conductance Regulator Function and Processing. J. Pharmacol. Exp. Ther..

[26] Billet, A., Froux, L., Hanrahan, J. W. & Becq, F. Development of Automated Patch Clamp Technique to Investigate CFTR Chloride Channel Function. *Front*. *Pharmacol*. **8** (2017). 10.3389/fphar.2017.00195 PMC5383655 28439239

[27] Meng X (2017). Two Small Molecules Restore Stability to a Subpopulation of the Cystic Fibrosis Transmembrane Conductance Regulator with the Predominant Disease-causing Mutation. J. Biol. Chem..

[28] Ma T (2002). Thiazolidinone CFTR inhibitor identified by high-throughput screening blocks cholera toxin–induced intestinal fluid secretion. J. Clin. Invest..

[29] Veit G (2014). Some gating potentiators, including VX-770, diminish F508-CFTR functional expression. Sci. Transl. Med..

[30] Cholon DM (2014). Potentiator ivacaftor abrogates pharmacological correction of F508 CFTR in cystic fibrosis. Sci. Transl. Med..

[31] Cholon DM, Esther CR, Gentzsch M (2016). Efficacy of lumacaftor-ivacaftor for the treatment of cystic fibrosis patients homozygous for the F508del-CFTR mutation. Expert Rev. Precis. Med. Drug Dev..

[32] Davies JC (2018). VX-659–Tezacaftor–Ivacaftor in Patients with Cystic Fibrosis and One or Two Phe508del Alleles. N. Engl. J. Med..

[33] Keating D (2018). VX-445–Tezacaftor–Ivacaftor in Patients with Cystic Fibrosis and One or Two Phe508del Alleles. N. Engl. J. Med..

[34] Collaco JM (2011). Effect of Temperature on Cystic Fibrosis Lung Disease and Infections: A Replicated Cohort Study. PLoS ONE.

[35] Collaco JM, Appel LJ, McGready J, Cutting GR (2018). The relationship of lung function with ambient temperature. PLOS ONE.

[36] Wang Chi, Aleksandrov Andrei A., Yang Zhengrong, Forouhar Farhad, Proctor Elizabeth A., Kota Pradeep, An Jianli, Kaplan Anna, Khazanov Netaly, Boël Grégory, Stockwell Brent R., Senderowitz Hanoch, Dokholyan Nikolay V., Riordan John R., Brouillette Christie G., Hunt John F. (2018). Ligand binding to a remote site thermodynamically corrects the F508del mutation in the human cystic fibrosis transmembrane conductance regulator. Journal of Biological Chemistry.

[37] Anderson MP, Welsh MJ (1991). Calcium and cAMP activate different chloride channels in the apical membrane of normal and cystic fibrosis epithelia. Proc. Natl. Acad. Sci..

[38] Schwiebert EM, Flotte T, Cutting GR, Guggino WB (1994). Both CFTR and outwardly rectifying chloride channels contribute to cAMP-stimulated whole cell chloride currents. Am. J. Physiol.-Cell Physiol..

[39] Linsdell P, Hanrahan JW (1996). Disulphonic stilbene block of cystic fibrosis transmembrane conductance regulator Cl-channels expressed in a mammalian cell line and its regulation by a critical pore residue. J. Physiol..

[40] Tousson A, Van Tine BA, Naren AP, Shaw GM, Schwiebert LM (1998). Characterization of CFTR expression and chloride channel activity in human endothelia. Am. J. Physiol.-Cell Physiol..

