# Supplementary material for: Short-term consequences of F508del-CFTR thermal instability on CFTR-dependent transepithelial currents in human airway epithelial cells
Source: Sci Rep. 2019 Sep 24;9:13729. doi: 10.1038/s41598-019-50066-7 (PMC6760155; doi:10.1038/s41598-019-50066-7)
Supplement: Supplementary file 1 — Supplementary information [file 41598_2019_50066_MOESM1_ESM.pdf]

Short-term consequences of F508del-CFTR thermal instability on CFTR-dependent transepithelial currents in human airway epithelial cells

Lionel Froux, Christelle Coraux, Edouard Sage, and Frédéric Becq

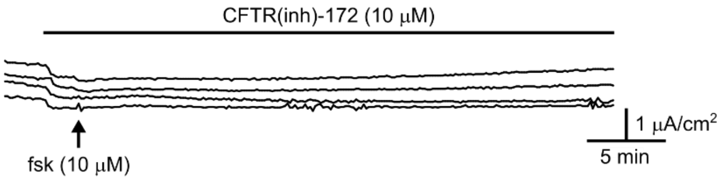

Supplementary Fig. S1: Stability of Ussing Chamber recordings in CFBE-F508del cells.

Representative Ussing Chamber traces obtained on CFBE-F508del cells after forskolin (10µM) addition. CFBE-F508del cells were not corrected with VX-809 and pre-incubated with CFTR(inh)-172 (10µM) for these experiments. fsk, forskolin.

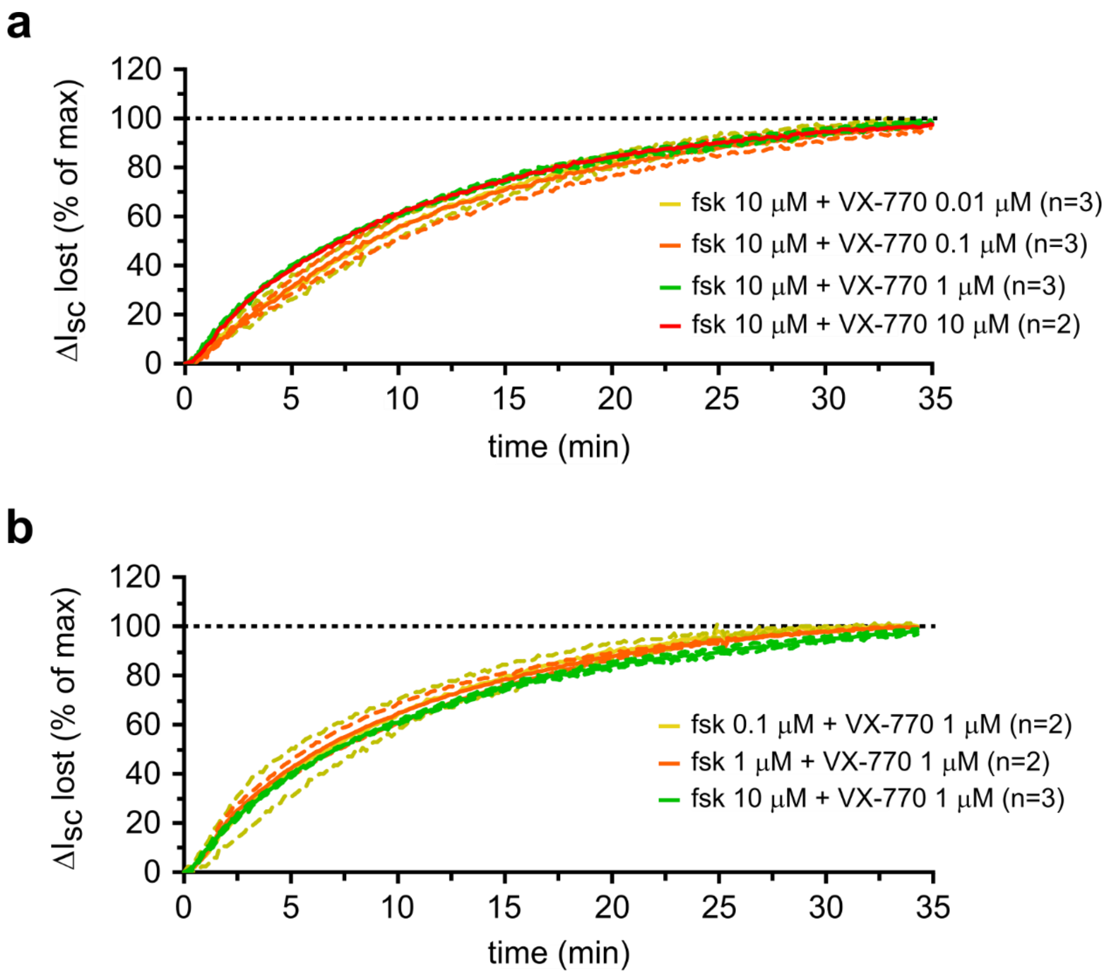

Supplementary Fig. S2: Changing VX-770 or forskolin concentrations does not impact temperature-dependent current loss rate.

a. Plot showing mean ΔI_{sc} lost expressed in % of maximal ΔI_{sc} lost against time using different concentrations of VX-770 (0.01, 0.1, 1 and 10µM) in addition to forskolin (10µM) to activate F508del-CFTR. n=2-3. **b.** Plot showing mean ΔI_{sc} lost expressed in % of maximal ΔI_{sc} lost against time using different concentrations of forskolin (0.1, 1 and 10µM) in addition to VX-770 (1µM) to activate F508del-CFTR. **a, b:** Data derived from Ussing Chamber recordings in CFBE-F508del cells corrected 24h with VX-809 (10µM). n=2-3. fsk, forskolin.

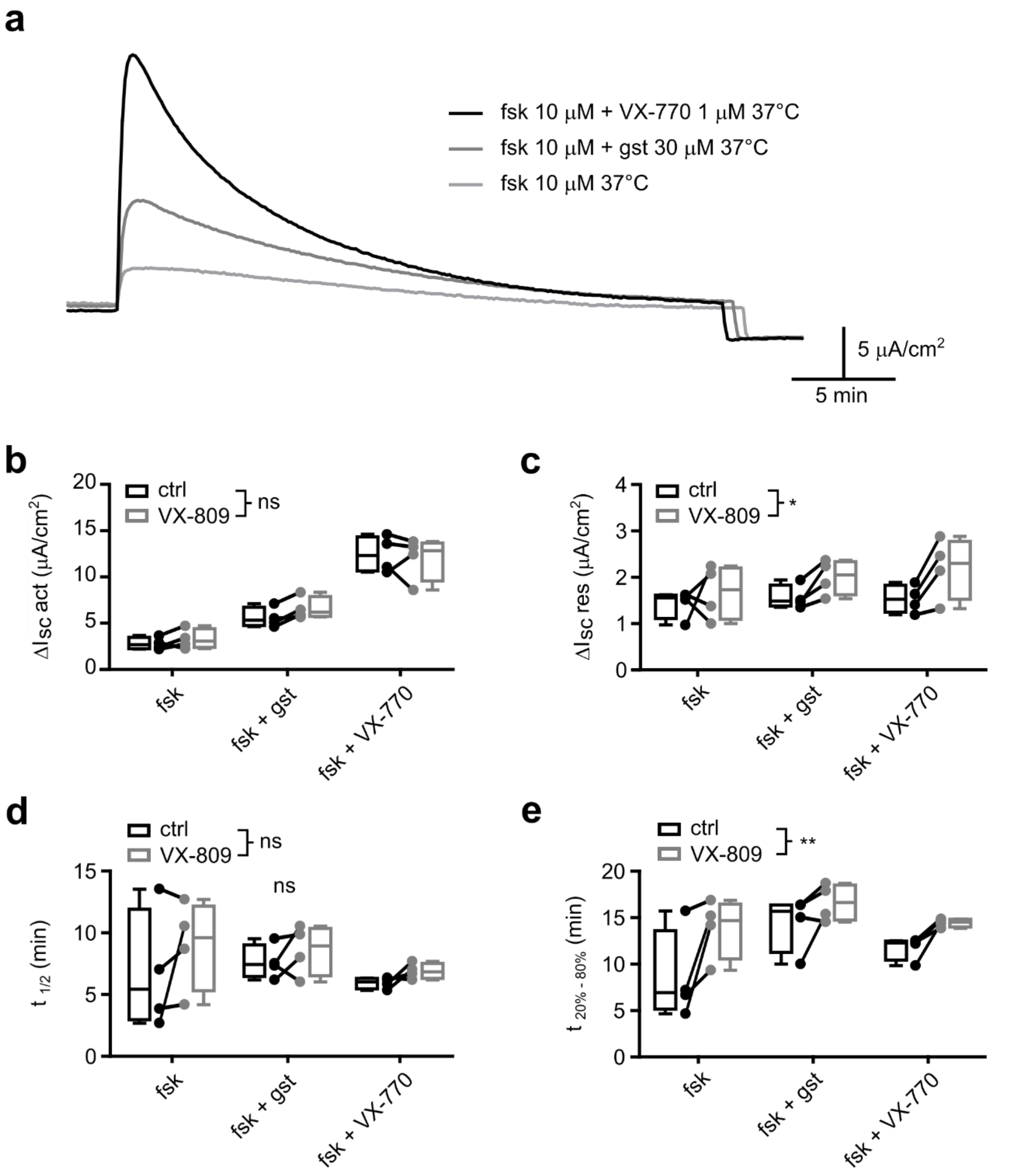

Supplementary Fig. S3: Acute VX-809 is not responsible for CFTR-dependent transepithelial current instability at 37°C in CFBE-F508del cells.

a. Ussing Chamber representative recordings obtained in CFBE-F508del cells at 37°C after forskolin (10 µM), forskolin + genistein (30 µM) or forskolin + VX-770 (1 µM) addition without acute addition of VX-809 (10µM) in the apical solution. CFBE-F508del cells were corrected for 24h with VX-809. **b-e.** Graphs displaying paired ΔI_{sc} act (**b.**), ΔI_{sc} res (**c.**), $t_{1/2}$ (**d.**) or $t_{20\%-80\%}$ (**e.**) values obtained without acute VX-809 (ctrl) and with acute VX-809 as well as their distribution, for forskolin, forskolin + genistein and forskolin + VX-770 conditions at 37°C. Recordings with and without acute VX-809 were systematically obtained on the same day on cells issued from the same passage for each condition. * $p<0.05$; ** $p<0.01$; ns, not significant; two-way repeated measures ANOVA (detailed results in supplementary table S5); n=4. ΔI_{sc} res, residual ΔI_{sc} 35 +/- 2 minutes after the peak or I_{sc} stabilization; ΔI_{sc} act, ΔI_{sc} after activation; fsk, forskolin; gst, genistein.

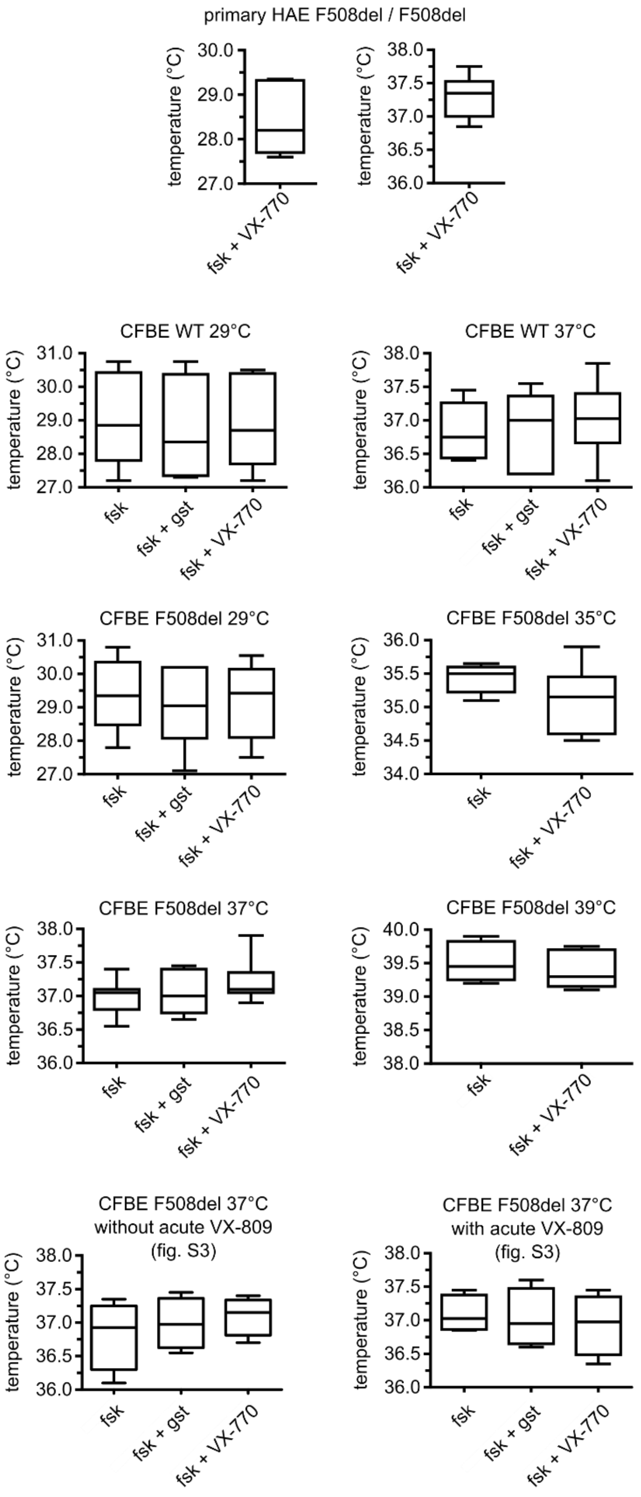

Supplementary Fig. S4: Temperature values recorded during Ussing chamber experiments.

Box plot representations displaying the distribution of the temperature values measured during experiments presented in this article. n=4-7. fsk, forskolin; gst, genistein.

	cell type	studied parameter	source of variation	F	p	p value summary
Fig. 3	CFBE WT	ΔI_{sc} act (Fig.3c)	interaction	2.90	0.094	ns
			treatment	0.22	0.81	ns
			temperature	15.91	0.0018	**
		ΔI_{sc} res (Fig.3e)	interaction	0.83	0.46	ns
			treatment	0.33	0.72	ns
			temperature	24.25	0.0004	***
	CFBE F508del	ΔI_{sc} act (Fig.3b)	interaction	23.92	< 0.0001	***
			treatment	13.75	0.0004	***
			temperature	195.2	< 0.0001	***
		ΔI_{sc} res (Fig.3d)	interaction	5.54	0.016	*
			treatment	33.26	< 0.0001	***
			temperature	56.56	< 0.0001	***
Fig. 5	CFBE F508del	$t_{1/2}$ (Fig.5b)	interaction	1.705	0.19	ns
			treatment	1.927	0.17	ns
			temperature	21.87	< 0.0001	***
		$t_{20\% - 80\%}$ (Fig.5c)	interaction	2.11	0.13	ns
			treatment	4.32	0.046	*
			temperature	222.9	< 0.0001	***
		ΔI_{sc} act (Fig.5d)	interaction	12.13	< 0.0001	***
			treatment	211.1	< 0.0001	***
			temperature	11.69	< 0.0001	***
		ΔI_{sc} res (Fig.5e)	interaction	32.74	< 0.0001	***
			treatment	64.62	< 0.0001	***
			temperature	79.92	< 0.0001	***
Supp. Fig. S3	CFBE F508del	ΔI_{sc} act (Fig. S3b)	interaction	1.484	0.2773	ns
			treatment	45.84	< 0.0001	***
			VX-809	1.066	0.3288	ns
		ΔI_{sc} res (Fig. S3c)	interaction	0.6204	0.5592	ns
			treatment	0.8631	0.4540	ns
			VX-809	8.683	0.0163	*
		$t_{1/2}$ (Fig.S3d)	interaction	0.3441	0.7178	ns
			treatment	0.6140	0.5624	ns
			VX-809	3.598	0.0904	ns
		$t_{20\% - 80\%}$ (Fig.S3e)	interaction	1.431	0.2886	ns
			treatment	2.993	0.1008	ns
			VX-809	17.52	0.0024	**

Supplementary table S5: Two-way ANOVA detailed results.

Table presenting detailed results obtained from two-way ANOVA analyses presented in Fig. 3, Fig. 5 and Supplementary Fig.3. “Treatment” refers to the way used to activate CFTR (forskolin, forskolin + genistein or forskolin + VX-770). ΔI_{sc} res, residual ΔI_{sc} 35 +/- 2 minutes after the peak or I_{sc} stabilization; ΔI_{sc} act, ΔI_{sc} after activation.
